# Standing Ready to Respond

**DOI:** 10.3201/eid3004.AC3004

**Published:** 2024-04

**Authors:** Byron Breedlove

**Keywords:** high-consequence pathogens, about the cover, art science connection, public health

**Figure:**
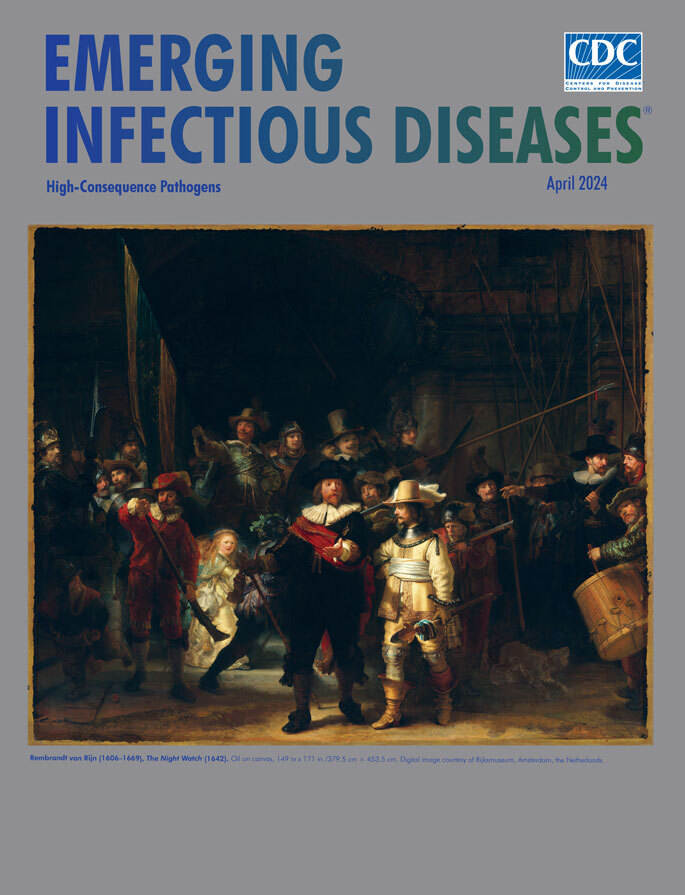
**Rembrandt van Rijn (1606–1669), *The Night Watch* (1642).** Oil on canvas, 149 in x 171 in/379.5 cm × 453.5 cm. Digital image courtesy of Rijksmuseum, Amsterdam, the Netherlands.

“Shine, shine, the light of good works shine.

The watch before the city gates, depicted in their prime.

The golden light all grimy now,

300 years have passed.

The worthy Captain and his squad of troopers standing fast.”

—King Crimson, “The Night Watch”

During a career spanning nearly five decades, Dutch artist Rembrandt van Rijn created approximately 350 paintings, 300 etchings, and 100 drawings. Rembrandt’s favorite subject was, apparently, himself. According to The National Gallery of Art, Washington, DC, USA, “nearly 80 self-portraits—paintings, drawings, and prints—are attributed to him.” Although those portraits chronicle his changing appearance as he aged, many other details of his life are lost. In her 2011 essay, “Much have I travel’d in the realms of gold,” Polyxeni Potter notes “Rembrandt’s life has been shrouded in mystery, largely because no written records exist beyond the usual certificates of birth, baptism, marriage, and death. He left no journal, and seven surviving letters from his hand concern routine transactions.”

That paucity of biographical details has not diminished Rembrandt’s reputation. The Rijksmuseum states that Rembrandt “is regarded as the greatest painter ever to have lived in the Netherlands. Paintings by him are currently valued at tens or even hundreds of millions of euros. The most famous of them all, *The Night Watch,* is estimated to be worth more than €500 million.” The title, however, is a misnomer. Art historians Zuzanna Stanska and Nicole Ganbold note, “For hundreds of years, the painting was coated with a dark varnish and dirt, which misled scholars into thinking that it depicted a nocturnal scene, hence its common title. In fact, throughout the centuries the layer of varnish grew so thick that it protected the canvas from a knife attack in 1911. The varnish was eventually removed in the 1940s, but the title remained.”

The actual title, *Officers and Other Civic Guardsmen of District II in Amsterdam, under the Command of Captain Frans Banninck Cocq and Lieutenant Willem van Ruytenburch*, seems too mundane for this nearly life-sized painting that has inspired several movies, the second movement of Gustav Mahler’s Symphony No. 7, a song titled *The Night Watch* by the band King Crimson, and a 2014 live reenactment by actors in a shopping mall in the Netherlands.

Rembrandt completed the painting in 1642, the year his wife Saskia died after a lengthy illness. The Rijksmuseum states that “Rembrandt's largest and most famous painting was made for one of the three headquarters of Amsterdam's civic guard. These groups of civilian soldiers defended the city from attack. Rembrandt was the first to paint all of the figures in a civic guard piece in action.” Yvette Hoitink, a professional genealogist, noted that such voluntary citizen militia, or *schutterij*, existed throughout the Netherlands from at least the 1500s until 1901 and could be summoned to quell riots and unrest, help control fires, and defend municipalities during war.

Rembrandt’s *The Night Watch* contrasts with static depictions of *schutterij* from the 1600s that show militia members standing in a row or seated around a banquet table. Art critic Fisun Güner wrote, “In this richly hued, tenebrous masterpiece, where light is used to lend the scene an ethereal quality amid the commonplace bustle of movement and action, we detect a certain strangeness, a certain unreality to the scene—even though it’s a painting full of noise.”

Appearing in the background before the city gates, shadowed figures are gesturing, conversing, and handling weapons; a standard bearer hoists a flag; and a drummer prepares to add his cadence. In this painting, Rembrandt shows, according to Güner, that “he is interested in creating a drama and bringing it to life with emotional force, mixing a sense of the solemn (or at least of attempted solemnity) and the comic. So here we have a ragtaggle crowd not quite managing to fall into step behind the figure of the captain as he gestures for his men to march out.”

In the foreground, Captain Banninck Cocq, garbed in black save a white frilled collar and red sash, issues orders. His lieutenant, Willem van Ruytenburch, listens to his captain, his luminous yellow-gold attire contrasting with the painting’s dark background. Near the center, the illuminated figure of a young girl seems out of context, but Stanska and Ganbold explain her role: “Attached to her dress we can see a dead chicken with its claws raised to the sky, a bag of gunpowder and firearms—all symbols of the guild. Rembrandt thought of her as an imaginary mascot of the civic militia.” The girl’s resemblance to the artist’s wife, Saskia, has also been noted. Rembrandt’s favorite subject also makes a guest appearance. Between the standard bearer and a helmeted militiaman, just behind the captain’s right shoulder, careful inspection reveals half the face of a man wearing a beret, identified by many as being Rembrandt.

The notion of a “night guard” to respond to situations that endanger the public resonates in other realms, including public health agencies that must be prepared to respond to an array of disease outbreaks and health threats. One priority is the more than 70 high-consequence pathogens: emerging and unknown bacteria, viruses, and prions, that can be easily transmitted from person to person, are associated with high mortality rates, and can potentially cause major public health outbreaks and trigger public fear.

Currently, the World Health Organization is “a leading a proactive approach to bolster global readiness and response to potential future epidemics and pandemics, the Research and Development (R&D) Blueprint maintains its commitment to expediting research on emerging disease threats.” That response includes the initiative dubbed “Disease X,” which the World Health Organization describes as a disease that could emerge and cause an international epidemic or the next global pandemic.

An effective, modern public health surveillance system to monitor disease outbreaks, collect information, and share findings is crucial. As Alexander Langmuir, the epidemiologist who started the Centers for Disease Control and Prevention Epidemic Intelligence Service, noted in his 1962 Cutter Lecture on Preventive Health, “Good surveillance does not necessarily ensure the making of the right decisions, but it reduces the chances of wrong ones.”

Planning for an outbreak from an as-yet unrecognized pathogen could help public health authorities respond more effectively with regard to detecting pathogen emergence; developing vaccines, treatments, and countermeasures; and recommending sound policies. Instead of starting from scratch, surveillance and planning would enable public health responders to be better prepared to venture forth, like the iconic figures immortalized in Rembrandt’s *Night Watch*, to ensure public health and safety.
